# Transcriptional Regulation of Osteoclastogenesis: The Emerging Role of KLF2

**DOI:** 10.3389/fimmu.2020.00937

**Published:** 2020-05-13

**Authors:** Daniela Rolph, Hiranmoy Das

**Affiliations:** Department of Pharmaceutical Sciences, Jerry H. Hodge School of Pharmacy, Texas Tech University Health Sciences Center, Amarillo, TX, United States

**Keywords:** rheumatoid arthritis, osteoclasts, KLF2, differentiation, transcriptional regulation

## Abstract

Dysregulation of osteoclastic differentiation and its activity is a hallmark of various musculoskeletal disease states. In this review, the complex molecular factors underlying osteoclastic differentiation and function are evaluated. The emerging role of KLF2 in regulation of osteoclastic differentiation is examined, specifically in the context of rheumatoid arthritis in which it has been most extensively studied among the musculoskeletal diseases. The therapies that exist to manage diseases associated with osteoclastogenesis are numerous and diverse. They are varied in their mechanisms of action and in the outcomes they produce. For this review, therapies targeting osteoclasts will be emphasized, though it should be noted that many therapies exist which bolster the action of osteoblasts. A new targeted molecular approach is under investigation for the future potential therapeutic development of rheumatoid arthritis.

## Introduction

Bone is a dynamic tissue which is constantly being remodeled. Its organic and inorganic components are formed by osteoblasts and degraded by osteoclasts. Bone critically contributes to systemic metabolic processes and regulation of blood calcium by acting as a reservoir, which can be liberated or stored as needed due to the specialized actions of osteoclasts and osteoblasts. Osteoclasts are specialized bone cells of myeloid origin that participate in skeletal turnover by resorbing bone. They are tissue-specific macrophage derivatives that exert their resorptive actions by carving out pits in bone via chemical secretions. Osteoclasts are critical to bone repair from micro-damage due to daily wear and tear and also play an important role in fracture healing (1). The dysregulation of their differentiation and activity, however, is a critical component of several diverse disease states of musculoskeletal origin. Impaired bone remodeling has varied and serious consequences, both locally and systemically.

Osteoclasts act in concert with osteoblasts and osteocytes, the other cells that make up bone tissue. Osteoblasts are bone-forming cells of mesenchymal origin that deposit the proteins and minerals that form the bone matrix. Osteoblasts modulate osteoclastic differentiation and activity by producing paracrine factors and other signaling molecules. Osteoblasts produce macrophage-colony stimulating factor (M-CSF) and receptor activator of NF-κB ligand (RANKL), key promoters of osteoclastogenesis (2). Moreover, they inhibit osteoclastic function by secreting osteoprotegerin (OPG) (3). Osteoblasts regulate osteoclasts via mechanisms distinct from the classical means of osteoclastic induction; they also exert their effects via the Wnt pathway, through Semaphorin 3A signaling, and IL-34, among others (4, 5). Osteocytes are bone resident cells; they are derived from osteoblasts that have become embedded in the matrix they produced. They are the most plentiful skeletal cells, comprising 90–95% of all bone cells. Osteocytes are important signaling cells; they produce soluble factors that regulate bone homeostasis (6). They respond to mechanical stress to induce bone remodeling by promoting osteoblast and osteoclast activity (7). They produce sclerostin, a secreted product that inhibits bone formation by osteoblasts (8). Moreover, they are capable of producing the cytokines that regulate osteoclastic differentiation and function.

## Osteoclast Origin and Differentiation

Osteoclasts are myeloid cells derived from hematopoietic progenitors. They originate from the fusion of preosteoclastic cells, becoming multi-nucleated cells in the process (9). Terminally differentiated osteoclasts secrete proteases and acids that mediate their resorptive activity. Osteoclastogenesis depends on two principal hematopoietic factors that are both necessary and sufficient for differentiation: RANKL and M-CSF, also known as colony stimulating factor (CSF-1) (10). Together, these promote expression of osteoclast-specific genes. Moreover, expression of a number of other genes exerts significant influence on the osteogenic process.

The RANKL/RANK/OPG axis critically regulates osteoclastogenesis (11). Developing and mature osteoclasts express RANK, a transmembrane signaling receptor responsible for activating downstream pathways that promote osteoclastic differentiation, activation, and survival. The ligand for this receptor is RANKL, a tumor necrosis factor (TNF)-related polypeptide that is secreted by osteogenic cells to promote *de novo* bone formation as well as remodeling. OPG is a secreted TNF receptor (TNFR)-related protein that is known as a decoy receptor for RANKL. RANKL binding to OPG instead of RANK forestalls osteoclastogenesis and bone resorption. A proper OPG/RANK ratio is critical for balanced bone remodeling (12).

## Osteoclast Function and Regulation

Osteoclasts participate in skeletal turnover by resorbing, or degrading, bone (13). They are polarized cells which create a microenvironment optimal for breaking down the organic and mineral structure of bone. Osteoclasts secrete hydrochloric acid (HCl), which functions to lower pH at the site of bone resorption. Furthermore, they secrete the protease cathepsin K, which works optimally at a lower pH (14). These chemical and enzymatic mechanisms form pits in bone, allowing for future deposition of new bone by osteoblasts.

Osteoclastic activity is essential for balanced bone remodeling (15). Bones constantly experience stress from the mechanical loads placed on them; osteoclasts aid in recovery from micro-damage. In the event of larger-scale damage, osteoclasts promote fracture healing to restore bones to full function. Bone is unique among tissues for its ability to recover from injury without the formation of scar tissue thanks to the orchestrated actions of osteoblasts and osteoclasts.

## Role of Reactive Oxygen Species in Osteoclastogenesis

At high concentrations, reactive oxygen species (ROS) can stress cells and bring about deleterious effects. At lower concentrations, however, they can serve as second messengers in various signaling pathways. ROS have been observed to activate mature osteoclasts to enhance bone resorption (16). Their role in osteoclastic cell development has likewise been demonstrated: ROS, including superoxide and hydrogen peroxide, have been identified as important mediators of the osteoclastogenic process, regulating expression of critical proteins such as p38 and JNK (17). Moreover, RANKL has been shown to generate ROS in osteoclastic precursors (18).

## Cellular Influence

Skeletal remodeling depends on the harmoniously orchestrated interplay between bone formation by osteoblasts and bone resorption by osteoclasts. Osteoclasts, therefore, develop in an environment populated by several other cell lineages and rely on signals from these cells to develop and function. As has been discussed previously, osteoblasts provide the signals for osteoclastogenesis in the form of secreted RANKL. Moreover, it is increasingly evident that immune cells play a critical role in osteoclastic differentiation, as osteoclasts and their precursors are sensitive to signals from pro- and anti-inflammatory cytokines. This contributes to the pathogenesis of several diseases and provides clues into how bone remodeling changes as people age.

In the past few decades, the role of immune influence on skeletal homeostasis has emerged as a prominent field of study. Chronic upregulation of inflammatory cytokines in individuals of advanced age, termed “inflammaging,” has been shown to influence bone health (19). During the process of bone resorption, there is intense cross-talk between osteoclasts and T cells. T cells secrete cytokines that promote osteoclastic differentiation and activity; furthermore, osteoclasts produce molecules that in turn activate T cells (20). This is especially important in diseases such as rheumatoid arthritis (RA) that have a well-established immune component, but is an important consideration in all diseases related to osteoclastic dysfunction.

## Osteoclasts' Role in Health and Disease States

Osteoclast dysfunction is a hallmark of a number of disease states. Osteoporosis is the most common metabolic disease, affecting over 10 million people over the age of 50 in the United States alone (21). Osteoporosis usually develops later in life and occurs when bone resorption by osteoclasts outpaces formation by osteoblasts, leading to fragile and brittle bones which are prone to fracture. Osteopetrosis is characterized by abnormally increased bone mass. It is caused by defects in osteoclast differentiation and function (22). It is a hereditary disease marked by mutations in a number of genes encoding proteins important to osteoclastic development and function. Other diseases that affect bone, such as osteoarthritis and bone metastases, are marked by inflammation that exacerbates osteoclastic activity (23, 24). While aberrant osteoclastic activity is implicated in many diseases, in-depth discussion of these diseases is beyond the scope of this review; we will here focus on RA, the impact of osteoclasts on disease progression, and the therapies that may help mitigate the destructiveness of this disease.

## Rheumatoid Arthritis

RA is an autoimmune disease characterized by inflammation and deterioration of small joints. Osteoclasts are the principal mediators of bone destruction in RA. These osteoclasts are activated by signals from T cells. Cross-talk between RANKL and IFN-γ has been shown to be critical for osteoclastic activation in RA (25). Inflammatory cytokines, especially TNF-α, IL-1, and IL-6, promote accelerated bone loss in RA. TNF-α is considered to be particularly important in RA pathogenesis, as it is a known inducer of RANKL and M-CSF expression (26, 27), yet also induces osteoclastic differentiation independent of RANK/RANKL (28). T helper cells can be sub-classified into Th1 and Th2 cells, distinguished by the cytokines they produce. Th1 cells produce IFN-γ and IL-2, while Th2 cells produce IL-4, IL-5, and IL-10. In RA, the ratio of these cell populations is skewed toward the Th1 phenotype, yet the RA synovium is characterized by nearly absent IFN-γ and IL-2 expression, indicating a dysfunction in these cells (29). The anti-osteoclastic activity of IFN-γ is therefore lost; furthermore, these defective Th1 cells can induce inflammatory cytokines, including RANKL, bringing about a microenvironment in which osteoclastic differentiation and activity are greatly increased.

## Rheumatoid Arthritis Therapy

Most of the treatments for RA focus on inhibiting the proliferation of aberrantly activated immune cells. This in turn affects osteoclasts indirectly, as they rely on signals from activated T cells for their own differentiation and activation. Treatment objectives for RA are to stop inflammation, alleviate symptoms, and prevent long-term damage to joints and organs. NSAIDs are used to relieve pain and inflammation associated with RA. Corticosteroids, including prednisone and its active metabolite prednisolone, have immunosuppressive properties and are therefore used to mitigate inflammation. Administered at low doses, they help patients manage pain and stiffness, while higher doses are prescribed to manage inflammatory flare-ups (30). Methotrexate is a common disease modifying anti-rheumatic drug (DMARD) with anti-proliferative and immunosuppressive properties (31). As a chemotherapeutic agent, it acts as an inhibitor of the enzyme dihydrofolate reductase and therefore halts thymine synthesis, preventing DNA replication and subsequent mitotic division. At the lower dose used to treat RA, it reduces the growth of rapidly dividing cells, including those of the immune system. The specifics of its mechanism of action in autoimmune diseases, however, are not entirely understood (32). TNF-α inhibiting therapies are used to attenuate inflammation in RA. These therapies have been shown to attenuate osteoclastogenic differentiation and activation by reducing B cell-surface RANKL expression as well as lowering serum levels of soluble RANKL (sRANKL) (33).

Despite numerous treatment options, RA remains a difficult disease to manage due to its complex immune underpinnings and degenerative nature. Hence, it is important to develop improved therapeutics which act via novel mechanisms. As will be discussed later in this review, KLF2 plays critical roles in regulating osteoclast differentiation and function and thus is a promising target for the development of new therapies.

## Regulation of Osteoclastogenesis

### Cytokines

Human osteoclastogenesis is regulated by two essential cytokines, M-CSF and RANKL. Moreover, it is affected by expression of pro-inflammatory cytokines, as the growing field of osteoimmunology reveals.

### Macrophage Colony Stimulating Factor

M-CSF is critical for the survival, proliferation, and differentiation of early osteoclastic precursor cells. It is produced by mesenchymal cells and their derivatives in the bone marrow microenvironment (34). Secretion of M-CSF is constitutive, yet regulated by several other factors. Following the withdrawal of estrogen after menopause, for instance, M-CSF levels increase due to increased circulating levels of the inflammatory molecules IL-1 and TNF-α (35). Furthermore, increased serum levels of parathyroid hormone promote elevated secretion of M-CSF (36).

**Figure 1:**
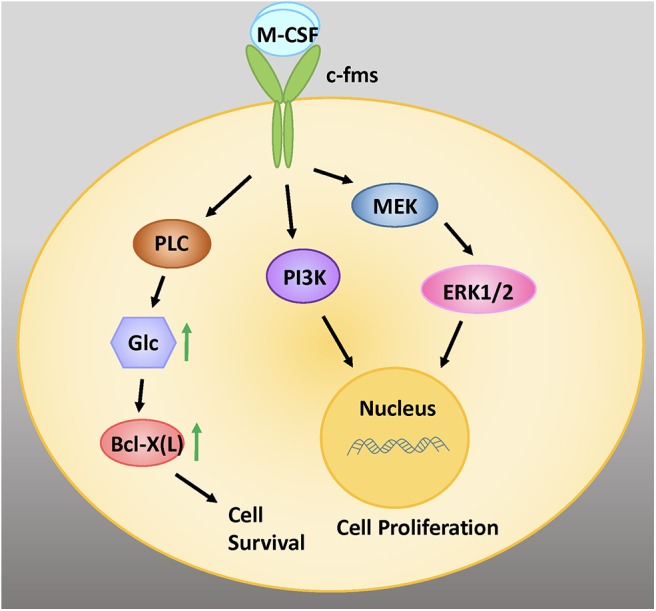
M-CSF signaling is critical for the survival, proliferation, and differentiation of early osteoclastic precursor cells. M-CSF binds to the cell surface receptor c-fms, a tyrosine kinase receptor. Intracellular signaling via the MEK/ERK and PI3 Kinase (PI3K) signaling pathways promote cell proliferation. Signaling via PLC increases intracellular glucose (glc), which increases levels of the anti-apoptotic protein Bcl-X(L), leading to cell survival. Adapted from Stanley and Chitu, *Cold Spring Harb Perspect Biol*, 2014 (37).

M-CSF binds to the cell surface receptor c-Fms, bringing about dimerization and tyrosine kinase activation (Figure 1) (38). Receptor autophosphorylation promotes downstream signaling that triggers a number of intracellular pathways, including extracellular signal-regulated kinases 1 and 2 (ERK1/2), phosphoinositide 3-kinase (PI3K), and phospholipase C gamma (PLCγ) signaling. Downstream signaling by mitogen activated protein kinases (MAPKs) including ERK has been shown to be instrumental for osteoclast development and function (39).

### Receptor Activator of NF-κB Ligand

Receptor Activator of NF-κB Ligand (RANKL) is instrumental for the terminal differentiation of myeloid cells into osteoclasts. RANKL binds to its transmembrane receptor RANK and induces intracellular signaling pathways, which include TRAF6 and c-Fos. These signaling cascades selectively activate nuclear factor of activated T cells, cytoplasmic 1 (NFATc1), a transcription factor critically important for expression of osteoclast-specific genes (40).

Osteoclastogenesis is regulated in part by the balance between RANK and OPG, the decoy receptor for RANKL. OPG is produced by osteoblasts and osteocytes and is either expressed on these cells' surfaces or secreted into the bone marrow space. OPG acts as a decoy receptor for RANKL, binding it and thereby preventing its interaction with RANK on pre-osteoclast cells (41).

### Transcription Factors

Following initiation of osteoclastogenesis by M-CSF and RANKL, a number of downstream pathways and gene regulation mechanisms are activated. Osteoclastic differentiation requires activation of transcription factors including microphthalmia-associated transcription factor (MITF), c-Fos, NF-κB, and NFATc1. These transcription factors promote the expression of genes critical for osteoclast phenotype and function. Recently, Kruppel-like factor (KLF) 2 has also emerged as an important regulator of osteoclastic differentiation and activity.

### Microphthalmia-Associated Transcription Factor

MITF regulates the development and activity of several cell lineages, including osteoclasts. The isoform MITF-E, which is significantly upregulated in developing osteoclasts but is virtually absent in macrophages, is induced by RANKL and has been shown to be critically important for osteoclastogenesis (42). Moreover, MITF-E has more recently been shown to regulate osteoclastogenesis by modulating the activity of NFATc1 (43). MITF is activated downstream of p38 MAP kinase, which in turn is activated as a result of RANKL signaling. MITF is crucial to the expression of genes encoding osteoclast-specific proteins tartrate-resistant acid phosphatase (TRAP) and cathepsin K (2).

### NFATc1, NF-κB, and c-Fos

**Figure 2:**
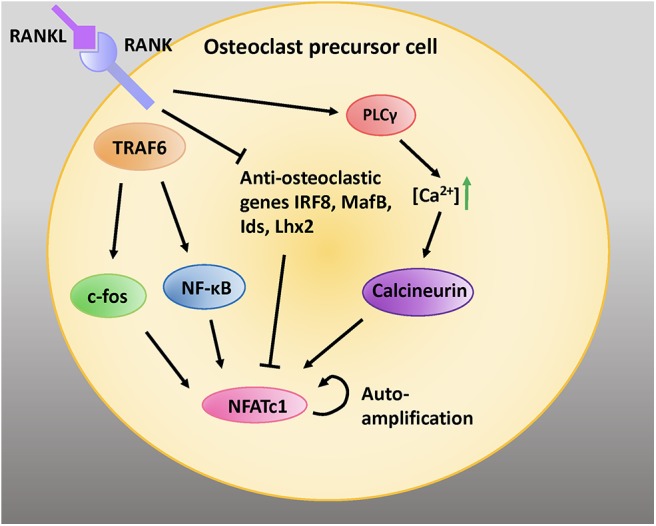
RANK/RANKL signaling is critical for osteoclastic differentiation and function. RANKL binding to RANK expressed on the cell surface of preosteoblastic cells activates intracellular adaptor protein TRAF6. Activation of c-fos and NF-κB pathways induces the NFATc1 gene. Moreover, activation of PLCγ increases intracellular calcium concentrations; this activates the protein calceineurin, which leads to recruitment of NFATc1 to its own promoter which further induces NFATc1. RANK signaling inhibits the anti-osteoclastic genes interferon regulatory factor 8 (IRF8), V-maf avian musculoaponeurotic fibrosarcoma oncogene homolog B (MafB), inhibitors of differentiation (Ids), and LIM homeobox 2 (Lhx2), which inhibit NFATc1 expression. Adapted from Kim and Kim, *J Bone Metab*, 2014 (45).

NFATc1 is a transcription factor that is amplified downstream of RANKL activation (11). RANKL signaling via TNF-receptor associated factor 6 (TRAF6) leads to auto-amplification of NFATc1, the master transcription factor regulating osteoclastogenesis (44). Intermediate signals downstream of TRAF6 are mediated via NF-κB and c-Fos (Figure 2) (45).

NF-κB is a family of transcription factors that mediate a number of cellular processes. Notably, NF- κB signaling is involved in inflammatory gene transcription; moreover, it is known to mediate RANKL induced osteoclastogenesis (46). In the absence of an activator ligand, the p50 and p65 subunits of NF-κB are inhibited by their interaction with the inhibitor of κB (IκB) protein, which prevents their nuclear translocation. In the “on” state, IκB is phosphorylated by IκB kinase (IKK), which marks it for polyubiquitination and subsequent proteasomal degradation (47). The free subunits of NF-κB then translocate to the nucleus where they modulate gene transcription. NF-κB signaling is initiated in cells by a variety of stimuli, including cytokines, immune modulators, and other factors. Intracellular signaling cascades are activated not only by RANKL but also by TNF-α, IL-1, and radical oxygen species (ROS), among others. The receptors for these molecules often associate with intracellular adaptor proteins such as TRAFs that facilitate recruitment of the downstream signaling molecules that bring about NF-κB activation. c-Fos is a proto-oncogene that critically regulates osteoclastogenesis (48). Like NF-κB, it is a transcription factor that is activated downstream of RANKL, TNF-α, and IL-1 signaling (49). Moreover, evidence suggests that c-Fos regulates RANK expression (50).

Given the importance of NF-κB in RA pathogenesis, it seems to be a sensible target for RA therapeutic development, however, none have been approved for use in patients (51). While it is important to keep exploring this trajectory, it is critical to investigate the therapeutic potential of emerging targets. Emerging findings show that KLF2 regulates most of the inflammatory genes that are regulated by NF-κB, therefore, next we focus on emphasizing the role of KLF2 in regulation of osteoclast differentiation and function in the context of RA and beyond.

### KLF2

#### KLF Structure and Function

Krüppel-like factors (KLF) are a family of DNA-binding zinc-finger proteins that can act as either transcriptional activators or repressors. Eighteen members of the KLF family have been identified so far. Their expression varies widely among tissues, pointing to the specific functions of each isoform (52). KLF family proteins are associated with development, metabolic regulation, and maintenance of homeostasis in diverse organs and tissues, from the heart to muscles to blood cells. Many are essential to life: global knockout of most KLF isoforms *in vivo* causes severe complications or death. KLF4 is especially well-known to researchers due to the role it plays in embryogenesis and for its groundbreaking utility in induced pluripotent stem cell reprograming (53–55).

KLF proteins play a significant role in physiological processes including cell differentiation, growth, and proliferation, as well as in responses to stressors such as apoptosis. Changes in their activity are associated with diverse pathologies, from cardiovascular disease to metabolic aberrations to cancer (56). They share homology with the transcription factor Sp1, which binds CG-rich regions of DNA with its zinc-finger structure; Sp1 and KLF are often classified as members of a common family. The Sp1/KLF family regulates the expression of genes with diverse functions in cell maintenance, development, and homeostasis (57).

#### KLFs in Bone Biology

Several KLF proteins are involved in bone health and disease. KLF5, expressed in osteoblasts and chondrocytes but not in osteoclasts, is associated with cartilage degradation. This process is mediated by increasing transcription of MMP9 (58). KLF15 has been shown to be upregulated by glucocorticoids, and in turn to impair osteoblast differentiation and bone formation (59). A recent publication demonstrates that KLF10 mediates chondrocyte hypertrophy during development (60). KLF4 expression increases in response to inflammatory stimuli in macrophages and mediates pro-inflammatory signaling pathways in these cells, which may have effects on bone biology given that osteoclasts are derived from myeloid precursors (61). KLF2, discussed in detail below, has been shown to play a critical role in osteoclast and osteoblast development and activity. Further investigation in the field may reveal skeletal involvement of other KLF isoforms.

#### Physiologic Roles of KLF2

KLF2, also known as lung KLF, is expressed during embryonic development in vascular endothelial cells and is upregulated in endothelial cells subjected to shear stress from prolonged laminar blood flow (62). Homozygous KLF2 knockout is embryonic lethal due to the critical role that KLF2 plays in promoting vessel integrity (63). KLF2 is best known for its importance in lung function, cardiovascular development, and for its atheroprotective qualities (64–66). Moreover, it is a critical regulator of blood cell development, as it promotes erythropoiesis and T cell trafficking (67, 68). Emerging evidence suggests that it also plays a key role in cell differentiation and cellular response to inflammatory stimuli. For instance, KLF2 expression in endothelial cells is suppressed by pro-inflammatory cytokines IL-1β and TNF-α, both of which contribute to the pathogenesis of atherosclerosis. Moreover, myeloid-specific KLF2 deletion results in spontaneous activation of pro-inflammatory myeloid cell activation (69). TNF-α inhibits KLF2 by activating NF-κB and histone deacetylases (HDAC) 3 and 4 (70). In turn, KLF2 has been shown to regulate NF-κB mediated activities, including hypoxia inducible factor (HIF-1α) transcription in monocytes (69, 71). While it does not affect p65 accumulation in the nucleus, KLF2 strongly inhibits its transcriptional activity (72, 73). KLF2 inhibits NF-κB activity via direct interaction with epigenetic regulator p300 and PCAF, which are essential co-activators of NF-κB-directed transcriptional activity (72, 74). Moreover, KLF2 overexpression inhibits intracellular pathways that depend on IL-1β signaling (75). More specifically in the context of RA, global deletion of KLF2 attenuates expression of MMP9 and inflammatory cytokines, contributing to elevated osteoclastogenesis and more aggressive disease progression (76).

#### Animal Models in KLF2 Studies

The development of animal models for the study of KLF2 is complicated by the fact that global knockout of the gene is embryonic lethal; absent KLF2 precludes proper formation of the vasculature in the developing pup (67). Endothelial KLF2 is essential for life at all stages, and endothelial deletion of KLF2 in adult mice is likewise lethal (77). For that reason, conditional KLF2 knockout in mice is a valuable tool to study the effects of this molecule. To overcome this challenge, two different knockout models are used in the studies detailed below: one is a hemizygous KLF2 knockout, in which the mice have a single copy of the gene instead of the two copies present in wild type animals (76, 78). The other is a monocyte-specific knockout characterized by normal expression of KLF2 in all tissues excluding the monocytes, which don't express any KLF2 (76).

K/BxN serum-induced RA is a useful model to study the immunologic forces at play in RA pathogenesis (79). To induce arthritis in this model, serum from arthritic transgenic K/BxN mice is injected into the foot pad of naïve mice. Signs of arthritis are evident within a few days of injection. The inflammation resulting from serum injection is caused by the formation of autoantibodies against endogenous glucose-6-phosphate isomerase, which leads to immune complexes that activate cells associated with the innate immune response. This reaction mimics the autoimmune nature of RA and this makes for a powerful model for the study of inflammation related to the progression of disease. Similar KLF2 dynamics are observed in K/BxN-induced RA models as well as in samples obtained from RA patients, indicating that this model accurately recapitulates the disease state in humans.

#### Emerging Roles of KLF2 in Bone Metabolism

**Figure 3:**
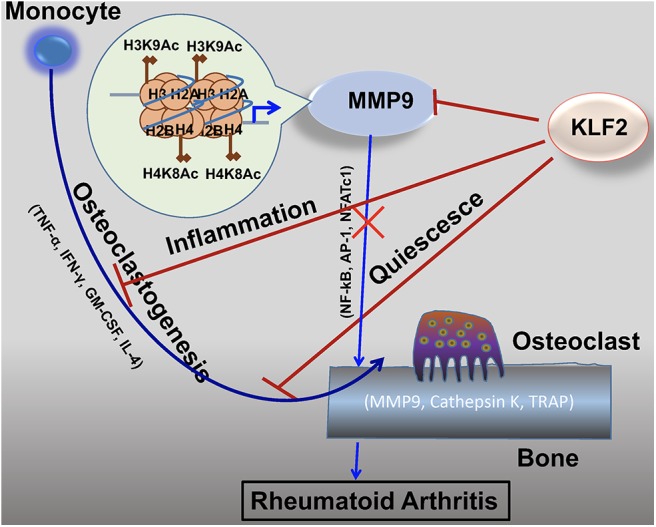
KLF2 attenuates osteoclastogenesis via several complementary mechanisms. KLF2 plays a role in epigenetic modulation of the pro-osteoclastogenic molecule MMP9; data from our lab show that KLF2 knockdown significantly increased enrichment of the active histone marks H3K9Ac and H4K8Ac and respective histone acetylase transfer (HAT) enzymes P300 and PCAF at the enrichment sites for the MMP9 gene (80). KLF2 inhibits pro-inflammatory markers, thereby further inhibiting monocytic activation (72). Furthermore, KLF2 promotes cell quiescence, decreasing osteoclastic activation and function. Altogether, its effects prevent excessive osteoclastic activity, slowing RA disease progression.

It is well-established that KLF2 inhibits pro-inflammatory activation of monocytes (Figure 3) (72). Moreover, studies investigating the effects of KLF2 in the context of RA show that, in mouse models of the disease, KLF2 modulates monocyte differentiation and function (78). In this study, KLF2 hemizygous mice were found to express higher levels of pro-inflammatory genes encoding monocyte chemoattractant protein 1 (MCP-1), cyclooxygenase-2 (COX-2), and plasminogen activator inhibitor-1 (PAI-1) than wild type (WT) controls. These molecules exert a pro-inflammatory influence in the context of RA by recruiting monocytes to the synovium, promoting angiogenesis in synovial tissue, and promoting fibrin accumulation in joint tissues, respectively (81–83). Moreover, methylated bovine serum albumin (mBSA) and IL-1β induced arthritis caused greater damage to cartilage and bones in KLF2 hemizygous mice compared to WT controls. In the RA models, higher recruitment of inflammatory monocytes to the joints was observed in KLF2 hemizygous mice compared to WT controls. Bone marrow-derived monocytes cultured *ex vivo* from KLF2 hemizygous mice underwent osteoclastic differentiation much more readily than cells taken from WT mice; moreover, the former osteoclasts possessed more aggressive pit-forming capacity, a measure of their function. Together, the data indicate that KLF2 plays an important role in attenuating inflammation and inhibiting osteoclastogenic differentiation and function in RA.

**Figure 4:**
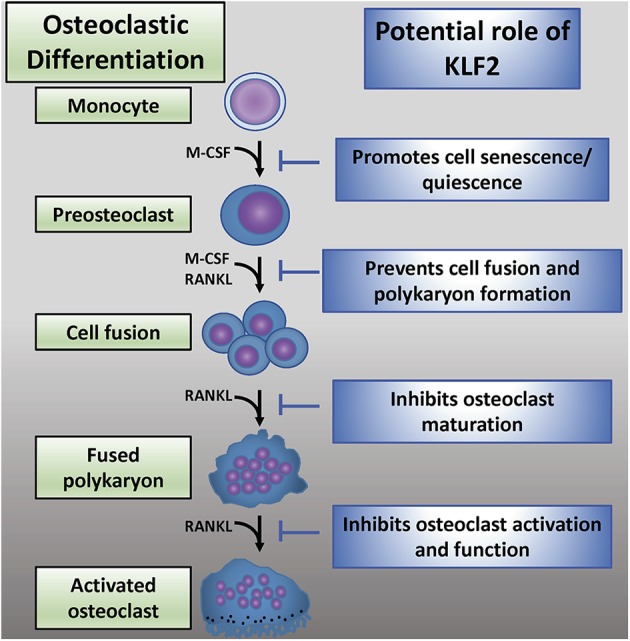
The emerging role of KLF2 in osteoclastic regulation. Osteoclastic differentiation from myeloid precursor cells is critically regulated by M-CSF and RANKL (2). KLF2 may play an inhibitory role at several stages of osteoclastic differentiation, maturation, and activation (72).

While KLF2 has a demonstrable role in osteoclastogenesis, the mechanisms governing this interaction are not well-understood (Figure 4). Recent reports detail work done in a K/BxN serum-induced model of rheumatoid arthritis which further illumines the role of KLF2 in regulating disease progression (80). In this study, RA induction was more pronounced in KLF2 hemizygous (KLF2^+/−^) mice compared to wild type (WT) animals; more ankle swelling and more severe immunohistochemical signs of inflammation and tissue damage were detected in the KLF2^+/−^ mice, whereas WT animals showed only mild signs of arthritic pathogenesis. After 7 days of arthritic induction, osteoclast precursor cells were isolated from the bone marrow of KLF2^+/−^ and WT animals and cultured *in vitro*. After 3 and 6 days of culture, osteoclastic differentiation was evaluated by TRAP staining; significantly higher numbers of TRAP stained cells were observed in the KLF2^+/−^ population compared to the WT population. Moreover, differentiated osteoclasts from the KLF2^+/−^ population expressed higher levels of the osteoclastic markers matrix metalloproteinase 9 (MMP9), NFATc1, and v-ATPase than WT cells. Expression of inflammatory molecules was also evaluated in cells isolated from the bone marrow of KLF2^+/−^ and WT mice: q-PCR revealed that cells obtained from KLF2^+/−^ animals express higher levels of the pro-inflammatory molecules IL-1β, IL-6, and TNF-α, higher levels of the chemokines CCL3 and MCP-1, and lower levels of the anti-inflammatory cytokine IL-10 than cells collected from the control group. These findings are consistent with the inflammatory nature of RA pathogenesis and demonstrate that KLF2 may play a protective role in bones and surrounding tissue by attenuating inflammation in arthritic joints.

In a subsequent study, K/BxN serum-induced arthritic induction was performed in a monocyte-specific conditional KLF2 knockout mouse model in order to determine whether KLF2 expression in monocytes is important for RA pathogenesis (76). Conditional KLF2^−/−^ mice showed pronounced ankle swelling which caused joint stiffness and impaired movement, whereas WT (KLF2^+/+^) mice showed minimal ankle swelling or distortion. At the tissue level, conditional KLF2^−/−^ mice showed more severe cartilage and subchondral bone degradation, as well as synovial thickening and hyperplasia; moreover, pannus formation and excessive production of fibrous tissue was observed, which is indicative of severe RA progression. To determine the role of monocytes in RA pathogenesis, bone marrow cells were isolated to study osteoclastogenesis and expression of inflammation-related markers. Increased expression levels of MCP-1, IL-1, IL-6, TNF-α, and MMP9 were observed in conditional KLF2^−/−^ mice compared to KLF2^+/+^ mice. In order to evaluate effects of monocyte-specific KLF2 knockout on osteoclastic differentiation and resorptive activity, bone marrow cells were harvested from conditional KLF2^−/−^ and KLF2^+/+^ mice after induction of arthritis. These cells were cultured *in vitro* in osteoclastic differentiation medium and stained for TRAP on days 3 and 6 of culture; much higher counts of TRAP+ cells were identified in the conditional KLF2^−/−^ mice compared to WT animals. Furthermore, osteoclasts were differentiated and cultured on ivory slices to evaluate their pit forming capacity; again, cells from conditional KLF2^−/−^ mice formed much more aggressive osteoclasts. Finally, MMP9 and MMP13 expression was evaluated in differentiated cells; expression of these molecules was significantly elevated in conditional KLF2^−/−^ mice relative to WT controls. These findings support the hypothesis that monocytic expression of KLF2 is critical for attenuating these cells' potential for osteogenic induction and inflammatory reactions.

In order to determine whether K/BxN serum-induced arthritis has any effects on KLF2 expression in monocytes, monocytes were isolated from C57BL/6 mice 7 days after induction of arthritis and q-PCR was performed to determine expression of various markers. Results indicated decreased KLF2 expression in both the bone marrow and in blood monocytes of arthritic mice relative to healthy controls. Furthermore, arthritic mice expressed increased levels of pro-osteoclastic markers TNF-α, MCP-1, MMP1, and MMP9. This indicates that KLF2 expression wanes over the course of arthritic induction, which allows for an increase in expression of pro-inflammatory proteins.

In human arthritic joints, immunohistochemical analysis was carried out to evaluate recruitment of activated (CD68^+^) monocytes to the inflammatory sites of RA tissues. More severe pathologic clinical manifestations were observed in human RA patients. Significant numbers of CD68^+^ monocytes were identified in all RA tissues evaluated in both the lining and sublining layers. Infiltration of numerous cell types was detected in inflamed RA tissues but not in healthy control tissues. q-PCR was carried out in order to evaluate expression of various molecules in peripheral monocytes in healthy vs. diseased patients. Importantly, this analysis revealed significantly decreased expression of KLF2 in monocytes obtained from diseased patients, as well as increased levels of the inflammatory factors TNF-α, MCP-1, MMP1, and MMP9. While a causal role is not established, these data suggest that a decreased level of KLF2 expression and concomitant increased levels of pro-inflammatory molecules are associated with RA pathogenesis not only in animal models of the disease but also in humans with symptoms of active RA.

It was observed that KLF2 knockdown increased MMP9 and NF-κB (p65) expression, and that KLF2 overexpression attenuated MMP9 and p65 levels. In order to better understand how KLF2 regulates MMP9, epigenetic studies were performed. Enrichment sites for epigenetic marks on the MMP9 promoter were identified and chromatin immunoprecipitation (ChIP) analysis and q-PCR were carried out. These experiments revealed that knockdown of KLF2 significantly increased enrichment of the active histone marks H3K9Ac and H4K8Ac and respective histone acetylase transfer (HAT) enzymes P300 and PCAF at the enrichment sites for the MMP9 gene. These findings indicate that KLF2 plays an important role in epigenetic modulation of the gene encoding MMP9. By inhibiting transcription of this gene, the destructive nature of osteoclasts is largely curtailed.

To test the therapeutic potential of KLF2, we have induced KLF2 using a pharmacological compound, histone deacetylase inhibitor (HDACi) and found that KLF2 expression was increased in myeloid cells both *in vitro* and *in vivo*. Induction of KLF2 significantly reduced osteoclastic differentiation of monocytes and decreased expression of matrix metalloproteinases in myeloid cells. Specifically in mice, induction of KLF2 in immune cells reduced arthritic inflammation and attenuated joint destruction. Furthermore, co-immunoprecipitation confirmed the direct interaction between KLF2 and HDAC4, thereby providing the groundwork to understand the molecular mechanisms whereby KLF2 regulates RA pathogenesis (80). Altogether, these data demonstrate a novel protective role of KLF2 in regulation of RA severity and progression.

Because KLF2 has demonstrable roles in regulation of RA pathogenesis, it is a promising therapeutic target. Moreover, further research into the pathways downstream of KLF2 activity may elucidate more signaling molecules that can be targeted to modulate disease progression and severity. Moreover, because KLF2 affects osteoclastogenesis, it may be a suitable target for a number of disease states associated with impaired osteoclast differentiation and function.

Similar findings have been reported by other labs showing that KLF2 overexpression in bone marrow-derived macrophages cultured in M-CSF and RANKL leads to lower rates of osteoclastic differentiation compared to control cells (84). Moreover, KLF2 overexpressing cells have lower mRNA expression of cFos, NFATc1, and TRAP and reduced levels of cFos and NFATc1 protein expression. Conversely, KLF2 downregulation with siRNA increases osteoclastic differentiation and leads to decreased expression of cFos, NFATc1, and RANKL mRNA as well as decreased levels of cFos and NFATc1 protein expression. They also found that KLF2 overexpression in primary osteoblasts cultured in osteoblastic differentiation medium containing BMP2, β-glycerophosphate, and ascorbic acid enhances their function by increasing expression of Runx2, alkaline phosphatase (ALP), and bone sialoprotein (BSP). Furthermore, KLF2 silencing decreases osteoblastic differentiation, as evidenced by significantly reduced ALP activity, bone nodule formation, and calcification, as well as decreased expression of osteoblastic marker genes compared to control cells.

This study also identified IFN regulatory factor 2 binding protein 2 (IRF2BP2), a regulator of KLF2, as a potential regulator of osteoclastogenesis and osteoblastogenesis. IRF2BP2 has been shown in several diverse research settings to attenuate inflammation; however, it has been studied less extensively than KLF2 (85, 86). In these studies, IRF2BP2 overexpression in osteoclast precursor cells significantly increases KLF2 expression, decreases osteoclastogenesis, and reduces expression of cFos and NFATc1 at the mRNA and protein levels and RANKL at the mRNA level. Moreover, IRF2BP2 overexpression in osteoblast precursors increases their expression of KLF2 and promotes osteoblastic differentiation and function by increasing expression of Runx2, ALP, and BSP. These findings indicate that IRF2BP2 mediates its effects on osteoblasts and osteoclasts by modulating KLF2. While IRF2BP2 overexpression inhibits osteoclastogenesis, its inhibitory effect is significantly reduced by silencing KLF2 with siRNA. In osteoblasts, IRF2BP2 overexpression increases differentiation, as evidenced by increased Runx2 and ALP expression and increased bone nodule formation; KLF2 silencing in these cells abrogates these effects. While the available data indicate that IRF2PB2 has a strong association with KLF2, the latter molecule appears to be more immediately important for the regulation of osteoclasts and osteoblasts. IRF2PB2 may be a promising anti-inflammatory target in the context of RA therapeutic development, however, much more is known about the role of KLF2 in this disease state. Moreover, it is not well-understood whether IRF2BP2 mediates osteoclast and osteoblast differentiation and function exclusively via KLF2 regulation or if it has effects independent of this downstream mediator.

A growing body of evidence supports the hypothesis that KLF2 regulates not only osteoclasts but also osteoblasts. A report indicates that KLF2 is expressed in osteoblast precursors as well as in mature terminally differentiated cells (87). This study found that osteoblasts cultured for 9 days in an osteogenic medium containing BMP2, β-glycerophosphate, and ascorbic acid expressed higher level of KLF2 throughout the differentiation process at both the mRNA and protein level. Moreover, cells cultured in osteogenic medium express higher levels of the osteoblastic marker genes ALP, osteocalcin, and osterix. Runx2 is a transcription factor that serves as the master controller for osteoblastic differentiation as well as the bone forming functions of mature osteoblasts (88). ALP, osteocalcin, and osterix genes are regulated by Runx2, which was also increased in expression throughout the differentiation process in a time-dependent manner at both the mRNA and protein levels. siRNA-mediated KLF2 knockdown led to reduced expression of Runx2, ALP, osteocalcin, and osterix in cells cultured in osteogenic medium, implying that KLF2 is a critical regulator of osteogenic differentiation. Moreover, KLF2 overexpression significantly enhanced Runx2, ALP, osteocalcin, and osterix expression at the mRNA and protein levels in osteoblastic progenitors cultured in osteogenic medium, further supporting the role of KLF2 in osteoblastic differentiation. KLF2 promotes osteoblastic activity by increasing expression of Runx2; importantly, immunoprecipitation experiments performed in HEK293T cells (human embryonic kidney cells) revealed that KLF2 physically interacts directly with Runx2 and thus promotes expression of ALP, osterix, and osteocalcin in osteoblast precursor cells. These findings illustrate the important role that KLF2 plays in promoting healthy skeletal remodeling and bone function.

Taken together, the data from our lab and those presented by other research groups provide a well-rounded view of the mechanisms by which KLF2 attenuates bone resorption and promotes bone formation. In the context of RA, curtailing the aggressiveness of osteoclasts is especially important, but the additional positive effects of KLF2 on osteoblasts indicate that this molecule may be a useful target in a number of diseases affecting the bones. Currently, most available medications aim to either reduce bone resorption or promote bone formation, but targeting KLF2 may lead to the development of therapies with dual functions. This emerging focus of study deserves the attention of researchers and holds great promise for future therapeutic developments.

## Conclusions and Future Directions

Osteoclasts play a critical role in human health and disease. They resorb bone in response to a number of factors secreted by resident bone cells as well as cells of immune system. They are necessary for the maintenance of skeletal health and maintaining homeostasis: insufficient activity leads to dense bones, while excessive activity results in brittle and fragile bones. While the role of osteoclasts in diseases such as osteoporosis is well-understood, how their dysfunction contributes to other diseases remains unknown. Moreover, certain targets represent untapped therapeutic potential; as new molecular pathways regulating osteoclasts are elucidated, new medications may be developed to treat osteoclast-related diseases. One such molecular target is KLF2: while its role in vascular development has been well-established, the mechanisms by which it regulates monocytes' differentiation into osteoclasts, as well as their activation and function are gradually being discovered. Moreover, their role in the promotion of osteoblastic differentiation and function further supports their utility as a target in bone metabolic diseases. Modulating KLF2 may prove useful to treat diseases involving osteoclasts.

## Author Contributions

Both authors contributed in writing manuscript and constructing figures.

## Conflict of Interest

The authors declare that the research was conducted in the absence of any commercial or financial relationships that could be construed as a potential conflict of interest.

## Abbreviations

ATP: Adenosine triphosphate
CCL3: chemokine ligand 3
ChIP: Chromatin immunoprecipitation
COX-2: Cyclooxygenase-2
CSF-1: Colony stimulating factor-1
DMARD: Disease-modifying anti-rheumatic drug
ERK: Extracellular signal-regulated kinase
HAT: histone acetylase transfer
HCl: Hydrochloric acid
HDAC: Histone deacetylase
HDACi: HDAC inhibitor
HSC: Hematopoietic stem cell
Ids: Inhibitors of differentiation
IFN-γ: Interferon gamma
IKK: IκB kinase
IRF2BP2: IFN regulatory factor 2 binding protein 2
IRF8: Interferon regulatory factor
IκB: Inhibitor of κB
JNK: c-Jun N-terminal kinase
KLF: Krüppel-like factor
Lhx2: LIM homeobox 2
MafB: V-maf avian musculoaponeurotic fibrosarcoma oncogene homolog B
MAPK: Mitogen activated protein kinase
mBSA: Methylated bovine serum albumin
MCP-1: Monocyte chemoattractant protein-1
M-CSF: Macrophage colony stimulating factor
MITF: Microphthalmia-associated transcription factor
MMP9: Matrix metalloproteinase
NFATc1, Nuclear factor of activated T cells: cytoplasmic 1
NSAID: Nonsteroidal anti-inflammatory drug
OPG: osteoprotegerin
PAI-1: Plasminogen activator inhibitor
PI3K: Phosphoinositide 3-kinase
PLCγ: Phospholipase C gamma
RA: Rheumatoid arthritis
RANK: Receptor activator of NFκB
RANKL: RANK ligand
ROS: Reactive oxygen species
sRANKL: Soluble RANKL
TNF: Tumor necrosis factor
TNFR: TNF receptor
TRAF: TNF-receptor associated factor
TRAP: Tartrate-resistant acid phosphatase
WT: Wild type.
